# Prevalence and determinants of contraceptive utilization among married women at Dabat Health and Demographic Surveillance System site, northwest Ethiopia

**DOI:** 10.1186/s12905-018-0611-3

**Published:** 2018-07-03

**Authors:** Geta Asrade Alemayehu, Abel Fekadu, Mezgebu Yitayal, Yigzaw Kebede, Solomon Mekonnen Abebe, Tadesse Awoke Ayele, Zemichael Gizaw, Mamo Wubeshet, Kindie Fentahun Muchie, Abebaw Addis Gelagay, Temesgen Azmeraw, Melkamu Birku, Kassahun Alemu, Amare Tariku, Terefe Derso, Adino Tesfahun, Nigusie Birhan Tebeje, Zemene Tigabu, Abebaw Gebeyehu, Getu Debalkie, Gashaw Andargie Biks

**Affiliations:** 1 Department of Health Service Management and Health Economics, Institute of Public Health College of Medicine, University of Gondar, Gondar, Ethiopia; 2 Department of Epidemiology and Biostatistics, Institute of Public Health College of Medicine and Health Science, University of Gondar, Gondar, Ethiopia; 3 Department of Human Nutrition, Institute of Public Health, College of Medicine and Health Science, University of Gondar, Gondar, Ethiopia; 4 Department of Environmental and Occupational Health and Safety, Institute of Public Health College of Medicine and Health Science, University of Gondar, Gondar, Ethiopia; 5 Department of Reproductive Health, Institute of Public Health College of Medicine and Health Science, University of Gondar, Gondar, Ethiopia; 6 Dabat Research Centre Health and Demographic Surveillance System, Institute of Public Health College of Medicine and Health Science, University of Gondar, Gondar, Ethiopia; 7 Department of Nursing, College of Medicine and Health Science, University of Gondar, Gondar, Ethiopia; 8 Department of Paediatrics, School of Medicine, College of Medicine and Health Science, University of Gondar, Gondar, Ethiopia; 9 Department of Health Education & Behavioural Science, Institute of Public Health College of Medicine and Health Science, University of Gondar, Gondar, Ethiopia

**Keywords:** Contraceptive utilization, Married women, Dabat HDSS, Northwest Ethiopia

## Abstract

**Background:**

Despite the enormous benefits of family planning services, the contraceptive utilization still remains low in Sub-Saharan Africa. There is regional variation in modern contraceptive utilization in Ethiopia. Therefore, this study was aimed to determine the prevalence of modern contraceptive utilization and determinants in Dabat demographic and health surveillance system site, northwest Ethiopia.

**Methods:**

A re-census was carried out in Dabat Health and Demographic Surveillance System (HDSS) site from October to December 2014. Data of 8271 married women collected in the re-census was used. The outcome variable was current utilization of any modern contraceptive methods whereas socio demographic and economic variables were the potential determinants considered. Bi-variable and multivariable binary logistic regression along with odds ratio and 95% confidence interval were used to describe the strength of association.

**Results:**

Prevalence of modern contraceptive utilization among married women in Dabat DHSS site was found to be 32.5% (95%CI: 31.5, 33.5%). After adjusting for covariates; the odds of using modern contraceptive were 2.35 times, 1.91 times, and 1.39 times higher among women of secondary and above educational level, urban residents, and women having six and above living children, respectively.

**Conclusion:**

Modern contraceptive utilization was found to be very low. Effort has to be applied to improve women’s educational level that increases their understanding of reproductive health issues. It is also important to give special emphasis for rural residents, those aged 20–40 years, and those with six or more living children while serving for modern contraceptive methods.

## Background

In 2015, world’s population reached 7.3 billion [1]. Population growth remains high in the group of 48 countries designated by the United Nations as the least developed countries (LDCs), of which 27 are in Africa [1, 2]. Ethiopia, the second most populous country in Africa, still has high fertility rate (4.6 children per woman) and fast population growth rate [3, 4]. The current projections showed a continued increase in population which requires strategic interventions [3, 5]. Ethiopia is one among the five most contributing to half of the world’s population growth during 2015–2050 [1, 2].

Family planning is widely approved as an important intervention towards achieving national and international goals as it has proven to reduce maternal and child mortality and morbidity [3]. It can prevent unwanted pregnancies and unsafe abortions [1, 2, 5, 6]. Family planning has also been found to promote gender equality as well as promote educational and economic empowerment of women [3, 5].

Despite the enormous benefits of family planning services, the utilization of contraceptives still remains low in Sub-Saharan Africa [1, 2, 5]. Besides, several factors including contextual, socio-economic, geographical or cultural may affect modern contraceptive utilization [7].

In Ethiopia, access of contraceptive service has been increasing due to the expansion of primary health care units (PHCU) [6]. However, it is still very low as compared to the national and international targets set at different times [3, 6]. The 2016 Ethiopian demographic and health survey report showed that the utilization for all types and long term methods of contraceptive was 35.3, and 10.3% respectively [4]. The most commonly used modern method is injectable, which is used by 31% of currently married women. However, only 5 and 3% of currently married women use implants and the pills respectively [3, 7]. This has resulted into high rates of unwanted pregnancies, unplanned deliveries and unsafe abortions resulting high maternal mortalities in the regions [3, 5, 6, 8].

There is regional variation in modern contraceptive utilization in Ethiopia [4, 9]. Studies conducted in Gondar town, northwest Ethiopia and North Showa zone, central Ethiopia reported contraceptive utilization of 48.4 and 46.9% respectively [8, 10]. However, studies addressing the proportion of women utilizing modern contraceptive and its associated factors are limited in the study area. Further, determining the prevalence and associated factors in the actual local setting are important to take appropriate and tailored interventions. Therefore, this study was aimed at determining the prevalence and associated factors of modern contraceptive utilization among married women of reproductive age group in Dabat demographic and health surveillance system (DHSS) site, northwest Ethiopia.

## Methods

### Study area and period

Dabat HDSS is one of full members of the International Network of Demographic Evaluation of Populations and Their Health (INDEPTH). Dabat HDSS site, located in northwest Ethiopia, currently includes a total of 13 kebeles (9 rural and 4 urban) with 17,000 households and 69,468 population. “Kebele” is the smallest administrative unit of Ethiopia, a neighborhood or a localized and delimited group of people. It is part of a woreda (district), itself usually part of a Zone, which in turn are grouped into one of the Regions based on ethno-linguistic communities that comprise the Federal Democratic Republic of Ethiopia. Each kebele consists of at least five hundred households, or the equivalent of 3500 to 4000 persons. The detailed profile describing establishment period, the setting, administrative organizational structure, population size, and objectives of Dabat HDSS are available at the website of University of Gondar [10].

The cross sectional re-census, including contraceptive utilization, carried out in October–December 2014 at Dabat HDSS was used for this study.

### Registration of contraceptive utilization

Dabat DHSS’s community surveillance includes house-to-house visits that contribute to an ongoing and continuous population events registration. In addition to the vital events, reproductive health issues including contraceptive utilization, pregnancy observation, antenatal care (ANC) attendance, place of delivery, maternal socio-demographic and other reproductive health characteristics were collected in the 2014 re-census. All married women in the study area were surveyed by trained fieldworkers. During the survey, each married women was asked about their ever and current contraceptive utilization status, type of contraceptives methods used, reasons for not using contraceptive methods and others contraceptive related issues.

### Variables of the study

Utilization of modern contraceptive methods including injectables, pills, implants, intrauterine contraceptive device (IUCD), condom, tubaligation and vasectomy, was considered as an outcome variable. A woman was taken as utilized if she is using any of the above modern contraceptive methods, whereas, others are taken as non-utilizers.

By reviewing related literatures [11–15] the variables age of the woman at first marriage, age at first birth, current age of the woman, place of residence, educational level, number of living children, religion of a woman and wealth of the household, were considered as a potential predictor variables in the study.

### Data management and analysis

The data were entered using Household Registration System (HRS) version 2.1 and exported to Stata version 14.1 for analysis. For the current analysis, we have included 8271 sexually active mothers in the reproductive age group (15–49 years) who are married in the study site during the study period.

Based on the highest education attended, women were classified into four categories: unable to read and write, able to read and write without formal education, primary educational level, and secondary/above educational level. Furthermore, principal component analysis (PCA) was used to classify women’s socio economic status. PCA used household assets, the number and kinds of consumer goods they own, ranging from a television to a bicycle or car, housing characteristics such as source of drinking water, toilet facilities, availability of electricity, and flooring materials.

The PCA scores were generated separately for the urban and rural household to consider wealth differences. The score for the household was derived from the dimension of the PCA explaining 69% the household variability. Finally, summed household common factor scores were assigned to each household member. Each member in the household population were ranked by their score, and divided into lowest, lower, middle, higher, and highest quantiles. Further, lowest and lower merged and taken as low income and higher and highest taken as better income.

Descriptive analysis, including proportions were performed. Binary logistic regression model was used to assess the association of various characteristics with modern contraceptive utilization. Bi-variable analysis was done to determine candidate variables for final multivariable analysis. Variables with *p*-value< 0.20) n bi-variable analysis were included in final multivariable binary logistic regression using enter method. Results were declared as statistically significant for *p*-values< 0.05. Adjusted odds ratio (AOR) with its respective 95% confidence interval (CI) were reported to show strength of association between utilization of modern contraceptive and explanatory variables.

## Result

### Socio demographic characteristics

The study result was based on 8271 sexually active married women at Dabat HDSS site. The predominant ethnicity and religion among these mothers in the Dabat HDSS site were Amhara, 8263 (99.9%) and Orthodox, 7983 (96.5%), respectively (Table 1). Six thousand four hundred forty four (77.9%) of the respondents were rural residents and 5258 (63.6%) of the respondent can’t read and write.

**Table 1:** Characteristics of participants in the study of contraceptive utilization at DHDSS, northwest Ethiopia, 2014

Characteristics	Number	Percent
Residence		
Rural	6444	77.9
Urban	1827	22.1
Religion		
Orthodox	7983	96.5
Muslim	288	3.5
Educational status		
Unable to read and write	5258	63.6
Able to read &write	711	8.6
Primary education	1186	14.3
Secondary and above	1115	13.5
Age		
< =20	1159	14.0
21–30	3254	39.3
31–40	2663	32.2
> =40	1195	14. 5
No of living children		
0–2	3054	36.9
3–5	2929	35.4
6+	2288	27.7
Age at first marriage		
< =10	1414	17.1
11–13	2049	24.8
14–17	3543	42.8
18 and above	1265	15.3
Age at 1st birth		
< 18	4132	50.0
18 and above	4139	50.0
Wealth index		
Low income	1429	17.8
Middle income	3502	43.7
Better income	3092	38.5

### Contraceptive utilization and reason for not utilizing

Overall prevalence of contraceptive utilization in Dabat DHSS site was found to be 32.5% (95%CI: 31.5, 33.5%). Majority of the contraceptive users, 2201 (81.3%) use injectable contraceptive methods followed by pills 228 (8.4) and implants 219 (8.1%) respectively (Table 2).

Desire to give birth (including those discontinued to give child birth) is the predominant reason, 1338 (28.6%) for not using the modern contraceptive methods followed by use of alternative methods (period cycle and breast feeding) 853 (18.2%), waiting for menstruation (including pregnant) 784 (16.7%), and fear of side effects 567 (12.1%) (Table 3). Here fear of side effects include those who discontinued because of side effects and related illnesses. However, the smallest share of the participants raised lack of access, religious and cultural prohibition, and lack of knowledge as reasons for not using modern contraceptive methods. Utilization is very low at rural residents (27.3%) and high at urban women (50.7%) (Table 2).

**Table 2:** Bi variable and multivariable logistic regression model for factors associated with contraceptive utilization at DHDSS, northwest Ethiopia, 2016

Variables		Currently using contraceptive		COR(95% CI)	AOR(95% CI)
		Yes (%)	No (%)		
Residence	Rural	1760 (27.3)	4684 (72.7)	1	1
	Urban	926 (50.7)	901 (49.3)	2.73 (2.45, 3.04)	1.91 (1.66, 2.20)
Education	Unable to read& write	1450 (27.5)	3829 (72.5)	1	1
	Able to read &write	219 (30.5)	498 (69.5)	1.16 (0.97, 1.37)	1.19 (0.99, 1.42)
	primary education	411 (34.6)	775 (65.4)	1.40 (1.22, 1.60)	1.42 (1.21, 1.65)
	secondary and above	606 (55.6)	483 (44.4)	3.31 (2.89, 3.78)	2.35 (1.92, 2.87)
Age of woman	<=20	842 (72.6)	317 (27.4)	1	1
	21–30	1205 (37.0)	2049 (63.0)	1.56 (1.34, 1.81)	1.32 (1.11, 1.58)
	31–40	918 (34.5)	1745 (65.5)	1.39 (1.20, 1.62)	1.33 (1.07, 1.65)
	> 40	246 (20.6)	949 (79.4)	0.69 (0.61, 0.78)	0.66 (0.51, 0.85)
No of living children	0–2	1075 (35.2)	1979 (64.8)	1	1
	3–5	985 (33.6)	1944 (66.4)	0.93 (0.83, 1.03)	1.30 (1.12, 1.50)
	6 and above	626 (27.4)	1662 (72.6)	0.69 (0.61, 0.78)	1.39 (1.15, 1.68)
Age at first marriage	<=10	369 (26.1)	1045 (73.9)	1	1
	11–13	580 (28.3)	1469 (71.7)	1.11 (0.95, 1.30)	1.13 (0.97, 1.33)
	14–17	1175 (33.2)	2368(66.8)	1.40 (1.22, 1.61)	1.25 (1.08, 1.45)
	18 and above	562 (44.4)	703 (55.6)	2.26 (1.92, 2.66)	1.10 (0.90, 1.35)
Age at first birth	< 18	1222 (29.6)	2910 (70.4)	1	1
	18 and above	1464 (35.4)	2675 (64.6)	1.30 (1.18, 1.42)	1.12 (1.00, 1.24)
wealth status	Lower income	933 (65.3)	496 (34.7)	1	1
	Middle income	2336 (66.7)	1166 (33.3)	0.93 (0.82, 1.06)	0.98 (0.85, 1.12)
	Higher income	2135 (69.0)	957 (31.0)	0.84 (0.73, 0.96)	0.88 (0.76, 1.01)

**Table 3:** Type of modern contraceptives utilized and reason for not utilizing at Dabat HDSS site, northwest Ethiopia, 2014

Characteristics	Frequency	Percent
Type of contraceptive method used		
Pills	228	8.4
Condom	11	0.4
Injectable	2201	81.3
Tuba ligation	14	0.5
IUCD	7	0.3
Implants	219	8.1
Other	26	1.0
Reason for not using the modern methods (*n* = 4684)		
To give birth	1338	28.6
Use alternatives	853	18.2
Waiting for menstruation	784	16.7
Fear of side effects	567	12.1
Stop birth naturally	393	8.4
Absence of sex	188	4.0
Lack of knowledge	178	3.8
Religious and cultural prohibition	150	3.2
Husband’s prohibition	120	2.6
Lack of access	90	1.9
Believe that natural spacing is ok	71	1.5

### Determinants of modern contraceptive utilization

Number of living children in the house hold, age of the woman, educational status, type of residence were significantly associated with modern contraceptive utilization in Dabat DHSS site.

A woman having six or more living children was 1.39 (AOR = 1.39, 95%CI: 1.15, 1.68) times more likely to use modern contraceptive as compared to woman with less than three children.

Women in the age group of 21–30 years were 1.32 (AOR = 1.32, 95%CI: 1.11, 1.58) times more likely to use modern contraceptives as compared to those less than or equal to 20. Besides, women in the age group of 31–40 were 1.33 (AOR = 1.33, 95%CI: 1.07, 1.65) times more likely to use modern contraceptives as compared to those less than or equal to 20 years. However, women in the age group of 40 or more were 34% (AOR = 0.66, 95%CI: 0.51, 0.85) times less likely to use modern contraceptives as compared to those less than or equal to 20.

Women at primary educational level were 1.42 (AOR = 1.42, 95%CI: 1.22, 1.65) times more likely to use modern contraceptives as compared to women who are unable to read and write. And women with secondary and above educational level were 2.35 (AOR = 2.35, 95%CI: 1.92, 2.88) times more likely to use modern contraceptives as compared to women who are unable to read and write. Urban women were 1.92 (AOR = 1.92, 95%CI: 1.67, 2.21) times more likely to use modern contraceptives as compared to their rural counterparts (Table 3).

## Discussions

Current use of contraceptive methods is one of the indicators most frequently used to assess the success of family planning programs. This study identified residence, mothers’ education, mother’s age, and number of living children as factors associated with current utilization of modern contraceptive methods in Dabat HDSS site, northwest Ethiopia.

The overall utilization of modern contraceptives in this study was found to be 32.5%. This finding is consistent with the United Nations millennium development goal (MDG) 2015 report for African continent (33.4%) [5], and the 2015 sub-Saharan MDG reports [5]. However, this finding is much lower than the findings of the 2016 Ethiopian DHS for Amhara region, 46.9% [4], the previous studies conducted in Gondar, Nekemte [14], Debreberhan [12] and west Gojjam [16]. The reason for this could be the fact that 78% of these study participants were from rural areas. It is also lower than the results from Ghana [17], Kenya [5], Nigeria [18] and Nepal [19]. However this result is higher than the results in Ghana [20], the United Nations MDG 2015 report for Somalia, Eritrea and South Sudan which was 23.7, 20 and 6.8% respectively [5]. The variations may be due to a different population characteristics and the countries health policy.

This study revealed that most of the contraceptive users (81%) were using injectable type of contraceptive methods. This predominant method was accounted in most previous studies [12–14, 21–24]. The reason may be due to its convenience of not being taken on daily basis and having comfortable ways of administration on top of the availability of this method than others.

In this study the proportion of long acting contraceptive utilization is found very low (8.62%). It is much lower than the findings conducted in Tigray region [15], in Nekemte [14], North Shewa [12], United Nations 2015 report for Ethiopia [5] and Burkina Faso [25]. However, the result is in line with the Ethiopian mini demographic and health survey (EMDHS) 2014 report [6], a study at Debretabore [26], the finding from west Gojjam [16], and that of south Sudan [5]. This variation may be due to the trainings given for health extension workers in some parts of the country to give long acting contraceptive methods and accessibility variations to health facilities for the service.

In this study, participants at the middle age group (20–40 years) were more likely to utilize modern contraceptive methods than women of age 20 or less and above 40 years. Contraceptive use of women with age group of 31–40 years was 66% more than women of age 41 to 49, and 35% more than the age group of 15–20 years. It is consistent with the studies conducted at different places of Ethiopia [13, 14]. This finding also coincides with the EMDHS 2014 results.

Educational attainment is found to be an important predictor of modern contraceptive use. In this study, women that had secondary education and above were more than two times to use modern contraceptive methods than those who couldn’t read and write. This agreed with the studies conducted in most developing countries [14, 16, 27, 28]. In this study, only 27% of women with no formal education report current use of any method, compared with 56% of women with secondary education and above. This can be explained by the notion that women with better educational level have better access to health care information, have greater autonomy to make decisions and have greater ability to use quality health care services [29, 30]. Moreover, the positive effect of education on contraceptive use could be associated with delay in marriages and first pregnancies, and increase women’s understanding of reproductive health issues like child spacing [27].

The present study revealed a significant difference in contraceptive use among the rural and urban residences. The odds of using contraceptive among women of urban dwellers are two times of their rural correspondent. The reality may be urban dwellers have better access to family planning services, and they are more likely to be better educated, and likely to use contraceptives more than the rural dwellers [11, 31].

Women who had six or more children were more likely to use modern contraceptives than those who had no children. The possible reason could be women who had more than 6 children may satisfy their demand to have more children. This finding is similar with previous reports from Zambia, Burkina Faso and Uganda which reported that as the number of living children increases, use of modern contraceptive increases [12, 26, 29, 32, 33].

## Limitations

This study was limited in assessing desired number of children, and overall knowledge and attitude towards contraceptives which can function as a barrier to contraceptive utilization [34]. However, the investigators believe that the mentioned limitations can’t inflict major impact on the validity of study findings.

## Conclusion

Modern contraceptive utilization is found to be very low. Residence, age of woman, education, number of living children are found significant predictors of modern contraceptive utilization at Dabat DHSS site. Effort has to be applied to improve women’s educational level that increases their understanding of reproductive health issues. It is also important to give special emphasis for rural residents, those aged 20–40 years, and those with six or more living children while serving for modern contraceptive methods.

## Abbreviations

ANC: Antenatal care
AOR: Adjusted odds ratio
CI: Confidence interval
COR: Crude odds ratio
DHSS: Demographic and Health Surveillance System
EMDHS: Ethiopian Mini Demographic and Health Survey
HRS: Household registration system
INDEPTH: International Network of Demographic Evaluation of Populations and Their Health
IUCD: Intra-uterine contraceptive device
LDCs: Least developed countries
PHCU: Primary Health Care Units
